# Transcriptome sequencing of *Festulolium* accessions under salt stress

**DOI:** 10.1186/s13104-019-4349-2

**Published:** 2019-05-31

**Authors:** A. Teshome, S. L. Byrne, T. Didion, J. De Vega, C. S. Jensen, M. Klaas, S. Barth

**Affiliations:** 1 Teagasc Crop Science Department, Oak Park, Carlow, R93XE12 Ireland; 2 DLF, Research Division, Store Heddinge, Denmark; 3 Earlham Institute, Norwich Research Park, Norwich, NR4 7UZ UK

**Keywords:** *Festulolium*, Transcriptome, Salt tolerance

## Abstract

**Objectives:**

The objective of this study was to establish transcriptome assemblies of *Festulolium* hybrids under salt stress, and identify genes regulated across the hybrids in response to salt stress. The development of transcriptome assemblies for *Festulolium* hybrids and cataloguing of genes regulated under salt stress will facilitate further downstream studies.

**Results:**

Plants were grown at three salt concentrations (0.5%, 1% and 1.5%) and phenotypic and transcriptomic data was collected. Salt stress was confirmed by progressive loss of green leaves as salt concentration increased from 0 to 1.5%. We generated de-novo transcriptome assemblies for two *Festulolium pabulare festucoid* genotypes, for a single *Festulolium braunii* genotype, and a single *F. pabulare loloid* genotype. We also identified 1555 transcripts that were up regulated and 1264 transcripts that were down regulated in response to salt stress in the *Festulolium* hybrids. Some of the identified transcripts showed significant sequence similarity with genes known to be regulated during salt and other abiotic stresses.

**Electronic supplementary material:**

The online version of this article (10.1186/s13104-019-4349-2) contains supplementary material, which is available to authorized users.

## Introduction

The *Festulolium* complex (FL) represents an array of interspecific hybrids between members of the genus *Lolium* and *Festuca* [1]. Breeders have been creating FL hybrids in order to combine the resilience of *Festuca* species against abiotic stresses with nutritive qualities and palatability of *Lolium* species [2–6]. Many FL hybrids are already in use including; *Festulolium pabulare*, *Festulolium braunii* (K. Richt.) A. Camus, *Festulolium brinkmannii* (A. Braun) Asch. & Graebn., and *Festulolium loliaceum* (Huds.) [4, 7, 8]. FL hybrids are increasingly utilised due to their resilience against high temperature and enhanced performance in cold and/or drought stressed environments [4, 9, 10]. Among the many challenges of modern agriculture, salt stress has become a serious threat as a consequence of irrigation, deforestation, land mismanagement, global warming and environmental pollution [11–13]. In Europe, ca. 3% of the arable land is affected by salinity of which coastal Southern Europe is the most affected due to seawater intrusion [12, 14]. And at the global level, ca. 30% irrigated arable land suffers from soil salinization [15].

This study describes the sequencing of the transcriptomes of three *Festulolium* hybrids (*F. pabulare fescuoid*, *F. braunii* genotype, and *F. pabulare loloid*) while under varying degrees of salt stress. We also use the gene models of *Lolium perenne* to compare gene expression under salt stress in the *Festulolium* hybrids. This study represents the first transcriptome assemblies of these *Festulolium* hybrids and is a resource for further studies.

## Main text

### Materials and methods

#### Plant materials and salt treatment

This salt tolerance experiment was carried out at Store Heddinge, Denmark during 2012/2013. A total of 16 FL hybrid varieties were included in the study (Additional file 1: Table S1). The 16 FL accessions consisted of: (1) four *Festulolium* spp. accessions, (2) four *Festulolium braunii* accessions with *Lolium multiflorum* × *Festuca pratensis* parentage, (3) four fescue phenotype like *F. pabulare* accessions with *Lolium multiflorum* × *Festuca arundinacea* parentage, and four ryegrass phenotype like *F. pabulare* accessions with *Lolium multiflorum* × *Festuca arundinacea* parentage. Seeds from each accession were germinated and grown in five replicates on 10 × 10 × 5 cm rock wool blocks and subjected to salt stress. The blocks were placed on tables that were intermittently flooded with water of the appropriate salt (NaCl) concentration and salt application was gradual. After 87 days establishment without salt, the plants were subjected to 0.5% NaCl for 28 days, then to 1.0% for 15 days, then to 1.5% for 34 days. Plant response to salt treatment was measured in terms of percentage of green leaves for each block with visual scoring. This phenotypic score was taken at three salt concentrations (0.5%, 1% and 1.5%). In order to ensure consistency, salt concentration was determined in terms of electrical conductivity in the solution (EC) which is a recommended methodology in similar scenarios (Additional file 2: Fig S1) [16].

#### Transcriptome sequencing

For RNA-seq analysis, leaves from four genotypes representing each of the three FL species [*F. braunii*, *F. pabulare fescuoid* (represented with two individuals) and *F. pabulare loloid*] were harvested at two time points, before (0% NaCl) and after salt treatment (1% NaCl) (Additional file 1: Table S1). Control leave samples were harvested at 87 days after sowing, right before salt treatment started. The second leaf sampling was carried out at 1% salt concentration (87 days 0% + 28 days 0.5% + 15 salt days 1.0%) (Additional file 2: Fig S1). Biological replicates were neither taken for control or treatment groups.

RNA was extracted with Qiagen RNeasy isolation kit. Quality control of RNA was carried out on a Bioanalyzer. Four libraries were sequenced per lane and all samples were sequenced on two lanes of an Illumina flow cell. Sequencing libraries were constructed with the TruSeq RNA sample preparation kit (Illumina) according to the manufacturer’s instruction, starting from 1.3 µg total RNA. After sequencing on the Illumina HiSeq 2000 platform, between 44 and 98 M paired-end (PE) reads per sample were obtained (Additional file 1: Table S1).

#### De novo transcriptome assembly and alignment

In total, 322.4 M PE reads of 125 bp were generated from all four FL individuals (GMAR040, GMAR053, GMAR055 and GMAR069) each with separate control and treated libraries. Raw sequence quality was assessed, for each library, using FastQC (v. 11.5) with default parameters [17]. Afterwards, Trimmomatic tool (v. 0.36) was used in order to trim adapter sequences and low quality bases [18]. A high quality reads with a Phred score of +30 and above and minimal length of 36 bases was kept for downstream analysis. After Trimmomatic filtering, a total of 304.3 M PE reads (ca. 94%), were retained for further analysis. Trimmed reads were assembled into unigenes/transcripts with the Trinity pipeline (v. 2.5.1) using default parameters [19]. An assembly for each individual was carried and quality of each assembly was checked by mapping reads back to the respective assemblies using bowtie2 (v. 2.2.9) [20]. In addition, the reads were also mapped against annotations of *Lolium perenne* genome (consisting of 40,068 transcripts) [21]. The completeness of each assembly was verified using BUSCO (Benchmarking Universal Single-Copy Orthologs) tool (v.3.0.2) (embryophyta odb9) [22]; to determine the presence of Embryophyta “near-universal single-copy orthologs”.

RNAseq reads from each individual sample were then aligned to the *Lolium perenne* annotations with Kallisto l [23] and transcript abundance was measured for sample. To identify genes regulated under salt stress in the FL hybrids, a single pairwise comparison was carried out between ‘control’ and ‘salt treated’ plants using Sleuth [24]. The control plants consist of two *F. pabulare fescuoid* genotypes, a single *F. braunii* genotype, and a single *F. pabulare loloid* genotype all at 0% NaCl at day 87. The salt treated plants consist of two *F. pabulare fescuoid* genotypes, a single *F. braunii* genotype, and a single *F. pabulare loloid* genotype all at 1% salt concentration (28 days 0.5% + 15 salt days 1.0%). Transcripts up/down regulated were identified using a likelihood ratio testing (LRT) and a Wald test. Transcripts identified as differentially regulated in both tests were retained and megaBLAST hit searches were carried out on them [25]. For up/down regulated transcripts, Gene Ontology (GO) enrichment analysis was completed with Blast2go program [26]. This program also carried out BLAST search and identified similarities of up/down regulated transcripts with other species.

### Results and discussion

#### Phenotypic characterization

**Fig. 1:**
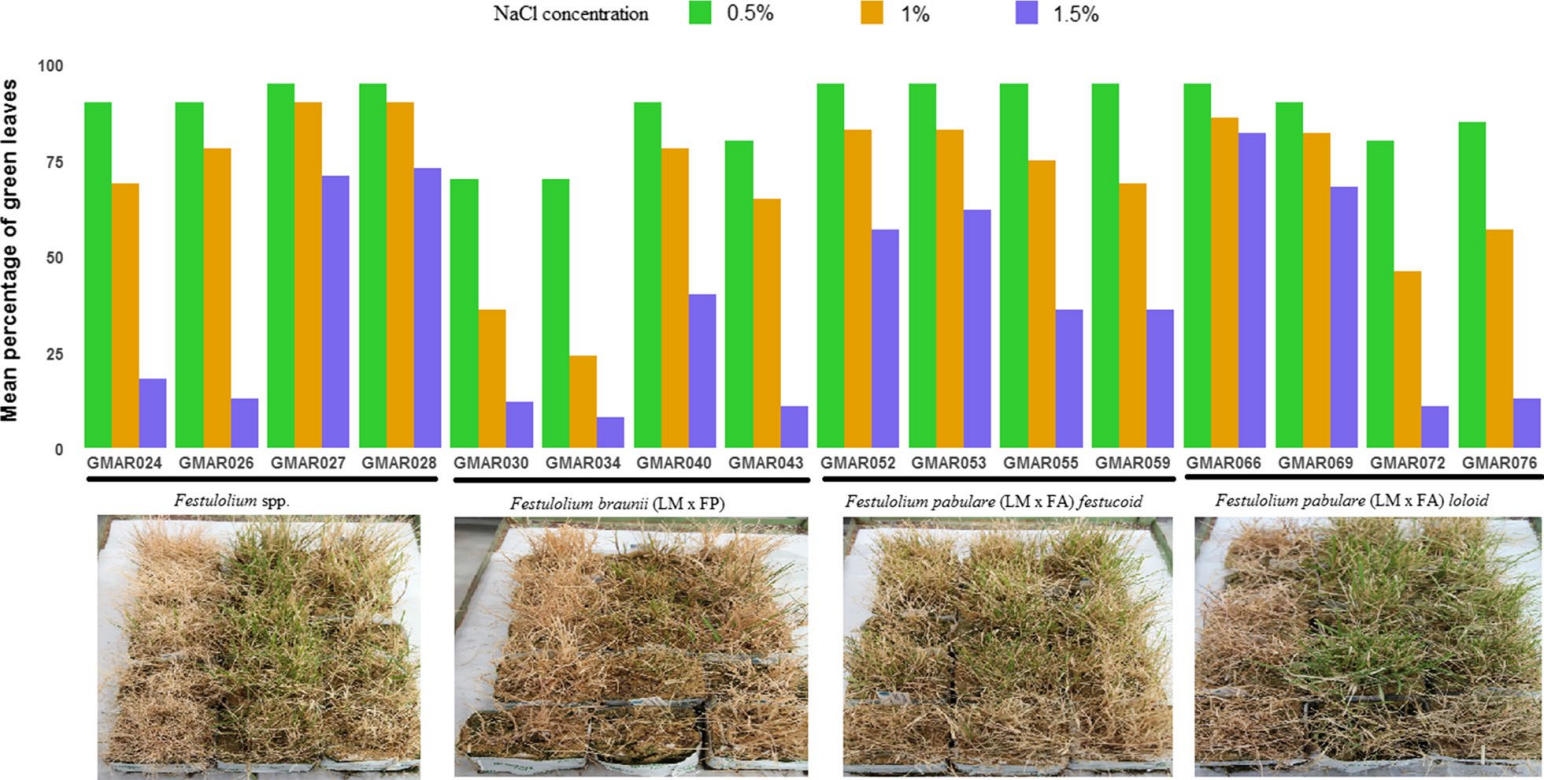
Reduction in mean percentage of green leaves with increasing salt concentration among different FL hybrids. Phenotypic characterization of FL hybrids under salt stress condition. This image was taken after 124 salt days for each block of hybrids

In general, salt stress has reduced the percentage of green leaves in all treated accessions (Fig. 1). A mild decrease in the percentage of green leaves was observed during the first period of salt treatment at 0.5% NaCl concentration for 28 days (Fig. 1). However, the reduction in green leaves percentage increased with increasing salt concentration. Previous studies indicated that salt stress disrupts photosynthetic machinery of cells preventing spatial and temporal leaf growth [27]. Among the four FL hybrids, the lowest green leave percentage was recorded for *Festulolium braunii* (< 50% green leaves at 1.5% NaCl concentration). Despite the general trend of loss of green leave percentage with increasing salt concentrations, genotypes like GMAR27 and GMAR28 (*Festulolium* spp.) and GMAR66 and GMAR69 (*F. pabulare*) recorded ca. 60% green leaves at the highest salt concentration (Fig. 1). Previous studies in perennial ryegrass (*Lolium perenne* L.) indicated that shoot parameters can predict salt tolerance [28] hence FL hybrids that performed well under aforementioned conditions can be potential candidates for breeding salt tolerant FL varieties.

#### Assembly of *Festulolium* hybrid transcriptomes

Control and stress reads for each of the four genotypes representing three *Festulolium* hybrids were pooled and used to generate four de novo transcriptome assemblies (Table 1).

**Table 1:** Statistics of de-novo assemblies for four *Festulolium* species

	GMAR040	GMAR053	GMAR055	GMAR069
	*F*. *braunii* (LM × FP)	*F. pabulare* (LM × FA) *festucoid*	*F. pabulare* (LM × FA) *festucoid*	*F. pabulare* (LM × FA) *loloid*
Total PE reads (10^6^)	42.5	83.9	84.4	93.5
GC (%)	49.1	47.9	48.7	48.9
All transcripts				
Total number of assembled bases (10^6^)	239.2	453.2	264.6	383
Average length	622	568	541	547
N50	872	739	707	704
Median length	406	383	360	371
Number transcripts	384,371	797,131	489,065	699,583
Number transcript clusters	194,108	378,661	246,789	338,190
Longest transcript in a cluster				
Total number of assembled bases (10^6^)	97.6	182.9	112.6	157.7
Average length	502	483	455	466
N50	616	573	524	542
Median length	320	320	300	311
Maximum transcript	15,832	17,601	13,519	14,268

Sequence reads were mapped back to their respective genotype specific de novo assembly with an overall alignment rate ca. 97% for all four assemblies indicating the majority of data has been assembled into contigs. The number of contigs in each of the four assemblies ranged between 384,371 and 797,131, corresponding to between 194,108 and 378,661 Trinity transcript clusters (Table 1). The longest isoform was between 13519 and 17601 bp among the assemblies. BUSCO v.3 [22] scores for complete genes (using the Embryophyta odb9 database) found in the respective assemblies ranged from 67.9 to 70.4%, indicating a high completeness (Additional file 3: Table S2).

#### Changes in transcript expression in response to salt-stress

A PCA distinguished between control and salt treated samples, with PC1 and PC2 explaining ca. 80% of the variation (Additional file 4: Fig S2). We grouped the salt treated samples together and compared them to the control samples to identify genes differentially expressed in *Festulolium* hybrids under salt stress. We used the published *Lolium perenne* gene annotations as a reference [21]. A total of 2819 transcripts were identified as differentially expressed between ‘control’ and ‘salt treated’ groups (Additional file 5). Among these, 1555 transcripts were up regulated, and 1264 transcripts were down regulated.

Among the up-regulated transcripts, maker-scaffold_5639|ref0010971-exonerate_est2genome-gene-0.3-mRNA-1, was highly up-regulated under salt stress in the FL hybrids (Additional file 5). This transcript shared significant sequence similarity with *Aegilops tauschii* subsp. *tauschii dehydrin* DHN2 (Accession: XM_020300071, 58% cov, 86% id,). *Dehydrin* transcripts were reported to accumulate in drought tolerant genotypes under water stress conditions [29]. Another up-regulated transcript, in ‘salt treated’ group, maker-scaffold_233|ref0040982-exonerate_est2genome-gene-1.0-mRNA-1 showed sequence similarity with *Brachypodium distachyon* plasma membrane H^+^-ATPase gene (Accession: XM_003561062.4, 74% cov, 93% id). This gene is reported to play key role in nutrients transport in general and in particular, its expression is expanded in Arabidopsis when grown in low phosphorus stress [30]. A third transcript, maker-scaffold_7832|ref0025452-exonerate_est2genome-gene-0.3-mRNA-3 which was up-regulated, in the treated group, showed significant sequence similarity with phospholipase D delta gene (Accession: NM_179170, 48% cov, 72% id). Arabidopsis phospholipase D delta gene mutants have shown drought tolerance traits in comparison to wild types under water stress conditions [31]. In general, a number of up regulated transcripts, in the present study, showed significant sequence similarity with well characterized abiotic stresses related genes and gene families.

**Fig. 2:**
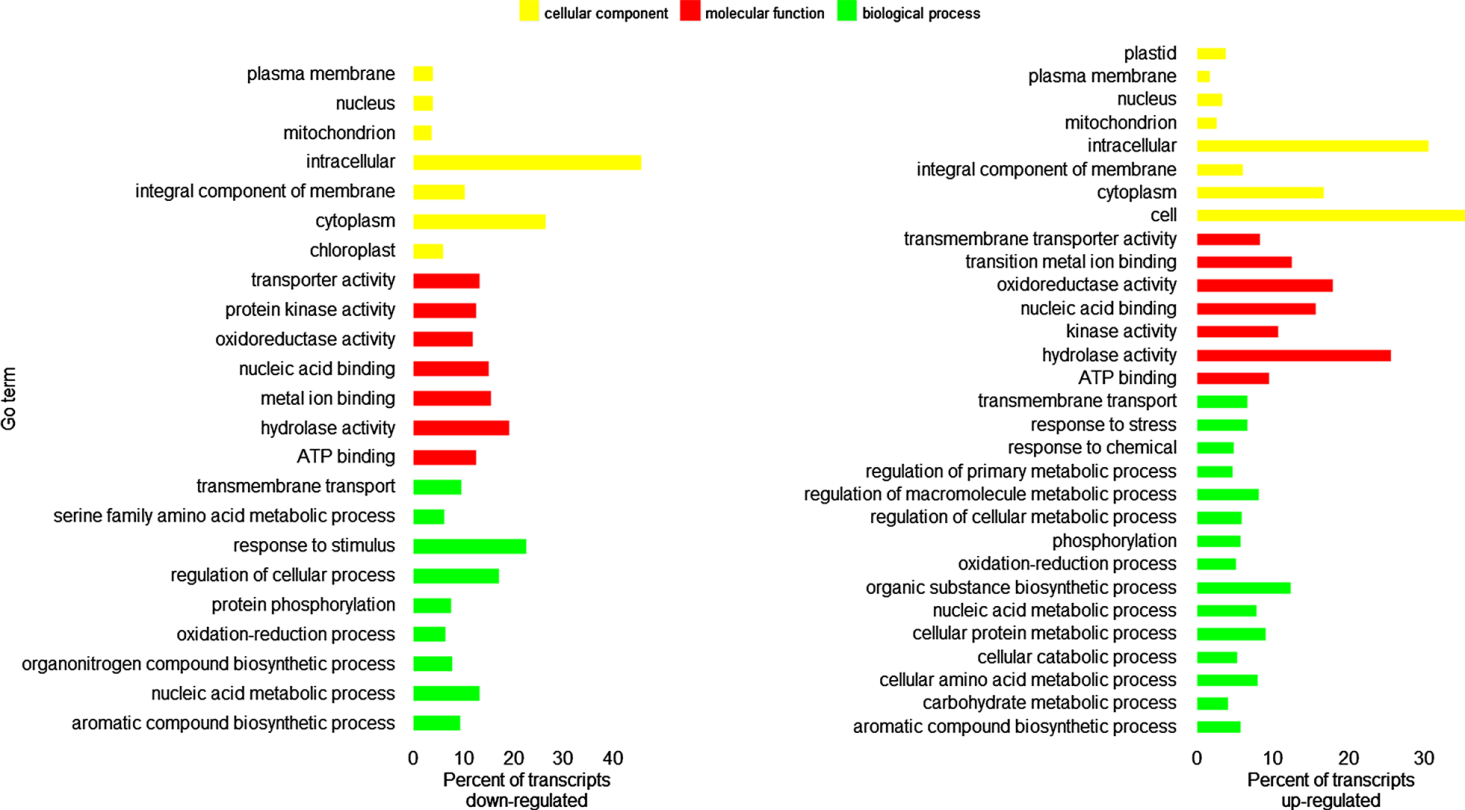
Diagram of Gene Ontology (GO) analysis output. **a** Up-regulated transcripts. **b** Down-regulated transcripts

Among significantly down-regulated transcripts, maker-scaffold_2836|ref0029302-exonerate_est2genome-gene-0.1-mRNA-6 showed sequence similarity with *B. distachyon* cysteine-rich receptor-like protein kinase 2 (Accession: XM_010236533.3, 87% cov and 92% id) (Additional file 5). Cysteine-rich receptor-like protein kinases are known to involve in stress response in many grasses and other species and its residues are known to cause cell death [32]. Another down regulated transcript maker-scaffold_2111|ref0018533-exonerate_est2genome-gene-0.0-mRNA-2 also showed significant BLAST hit (72% cov and 86% id) with *Aegilops tauschii* subsp. *tauschii* lipoxygenase mRNA (Accession: XM 020325112.1). This gene is reported to play key roles in different aspects of plant physiology like growth, development and resistance to pest/pathogen [33]. Another down regulated transcript, maker-scaffold_4704|ref0007237-exonerate_est2genome-gene-0.2-mRNA-1 showed sequence similarity with *Dasypyrum villosum* glutathione *S*-transferase (GST) mRNA (Accession: EU070904, 81% cov and 91% id). GSTs are known to be expressed at different plant developmental stages and also contribute towards wide range of abiotic stresses like salt, drought and temperature [34]. Both up and down regulated transcripts were annotated with the Gene Ontology (GO) terms and prediction function of the closest homologous. Accordingly, the most represented GO terms were quantified for each of the three GO categories, “biological process”, “cellular function”, and “molecular function” (Fig. 2). The highest proportions of up-regulated transcripts were associated with hydrolase activity and oxidoreductase activity “molecular function” terms. These enzymatic activities are already known to play roles in salt tolerance in different species [35, 36]. However, as leaf sample collection time point between ‘control’ and ‘salt treated’ groups was different, further sequencing and annotation of FL transcriptome is needed to characterize these transcripts and better understand salt stress-related pathways in FL species. The top three species on the basis of the best sequence alignment to each FL query are: (1) *Brachypodium distachyon*, (2) *Hordeum vulgare* subsp. *vulgare*, and (3) *Aegilops tauschii* (Additional file 6: Fig S3).

### Conclusion

In general, increasing salt concentration resulted in a loss of green leaves, confirming that plants were under salt stress. This enabled us to generate de-novo transcriptome assemblies for three *Festulolium* hybrids capturing genes expressed in response to salt stress. Furthermore, we identified a catalogue of transcripts regulated in *Festulolium* hybrids in response to salt stress. This represents the first transcriptome assemblies of *Festulolium* hybrids and the first look at transcriptional response to salt stress in *Festulolium* hybrids.

## Limitations

Differential expression is limited to identifying genes regulated across the *Festulolium* hybrids as we do not have sufficient replication to identify genes differentially expressed in each hybrid.

## Additional files

**Additional file 1: Table S1.** List of accessions used for phenotypic salt stress characterization and RNA-seq analysis.

**Additional file 2: Fig. S1.** Salt concentration during the experiment was measured in terms of electric conductivity of solution (EC). Control RNA samples were harvested at 0%NaCl concentration (87 days after sawing) and treatment RNA samples were harvested at 1%NaCl concentration (28 days 0.5% + 15 salt days 1.0%).

**Additional file 3: Table S2.** Summarized benching marking for each transcriptome assembly (C: complete [S: single, D: duplicated], F: fragmented, M: missing.

**Additional file 4: Fig S2.** PCA grouping between ‘control’ and ‘salt treated’.

**Additional file 5.** List of transcripts that passed both Wald and LRT pairwise comparison between ‘control’ and ‘salt treated’ groups.

**Additional file 6: Fig S3.** Top hit species distribution on the basis of sequence alignments and lowest E values.

## Data Availability

The transcriptomic data have been deposited: ArrayExpress accession E-MTAB-7720.

## Abbreviations

BP: base pair
BUSCO: Benchmarking Universal Single-Copy Orthologs
COV: coverage
FL: *Festulolium*
GST: glutathione *S*-transferase
ID: identity
LRT: likelihood ratio testing
PCA: principal component analysis
PE: paired end
