# Supplementary material for: Transcriptome sequencing of Festulolium accessions under salt stress
Source: BMC Res Notes. 2019 May 31;12:311. doi: 10.1186/s13104-019-4349-2 (PMC6545024; doi:10.1186/s13104-019-4349-2)
Supplement: Supplementary file 1 — Additional file 1: Table S1. List of accessions used for phenotypic salt stress characterization and RNA-seq analysis. [file 13104_2019_4349_MOESM1_ESM.pdf]

Entry No.	Name	Species	Time point for RNA sampling and total raw PE reads (10^6)
GMAR024	2005-40/9818	*Festulolium* spp.	
GMAR026	FL-5V2N 1/03	*Festulolium* spp.	
GMAR027	FLNHK08 H.V. Km11	*Festulolium* spp.	
GMAR028	FLNHK08 H.V. Km22	*Festulolium* spp.	
GMAR030	Achilles 1	*Festulolium braunii* (LMxFP)	
GMAR034	FLB 5-01-23384	*Festulolium braunii* (LMxFP)	
GMAR040*	HZ3/III 2005	*Festulolium braunii* (LMxFP)	0% and 1% (44.3)
GMAR043	PERSEUS ZIV 06	*Festulolium braunii* (LMxFP)	
GMAR052	FPF-5-07-22851	*Festulolium pabulare* (LMxFA) *festucoid*	
GMAR053*	FPF-5-07-22863	*Festulolium pabulare* (LMxFA) *festucoid*	0% and 1% (90.1)
GMAR055*	FPF-5-08-1105	*Festulolium pabulare* (LMxFA) *festucoid*	0% and 1% (89.1)
GMAR059	Korina 1	*Festulolium pabulare* (LMxFA) *festucoid*	
GMAR066	FELINA	*Festulolium pabulare* (LMxFA) *loloid*	
GMAR069*	FLIP-5-05-22810	*Festulolium pabulare* (LMxFA) *loloid*	0% and 1% (98.8)
GMAR072	FPL-05-11-11028	*Festulolium pabulare* (LMxFA) *loloid*	
GMAR076	Lofa 1	*Festulolium pabulare* (LMxFA) *loloid*	

*Accessions selected for RNA-seq analysis.
