# Supplementary figures and images for: Transcriptome sequencing of Festulolium accessions under salt stress
Source: BMC Res Notes. 2019 May 31;12:311. doi: 10.1186/s13104-019-4349-2 (PMC6545024; doi:10.1186/s13104-019-4349-2)

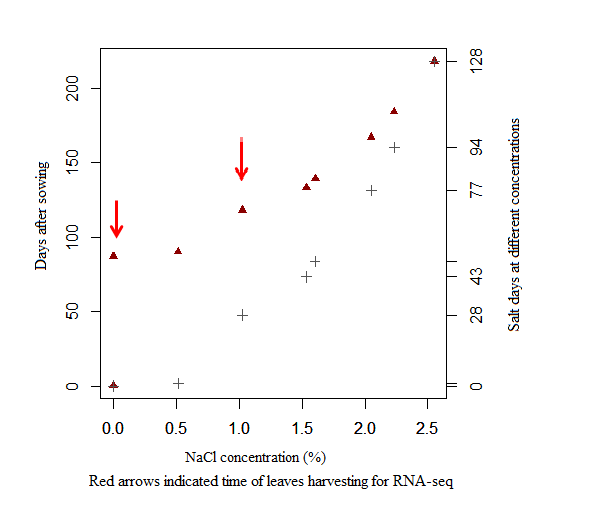

Supplement: Supplementary file 2 — Additional file 2: Fig. S1. Salt concentration during the experiment was measured in terms of electric conductivity of solution (EC). Control RNA samples were harvested at 0%NaCl concentration (87 days after sawing) and treatment RNA samples were harvested at 1%NaCl concentration (28 days 0.5% + 15 salt days 1.0%). [file 13104_2019_4349_MOESM2_ESM.png]

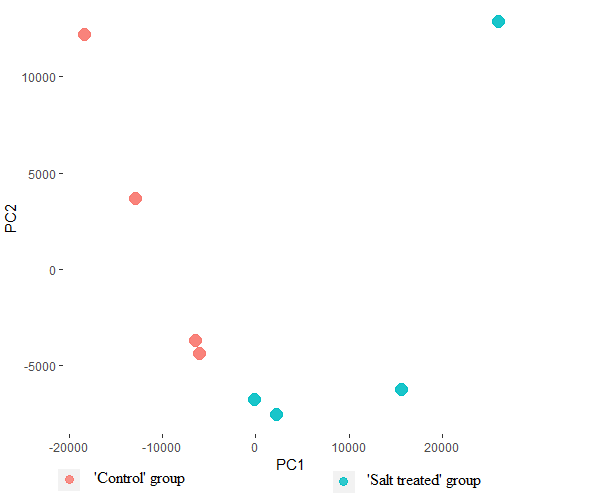

Supplement: Supplementary file 4 — Additional file 4: Fig S2. PCA grouping between ‘control’ and ‘salt treated’. [file 13104_2019_4349_MOESM4_ESM.png]

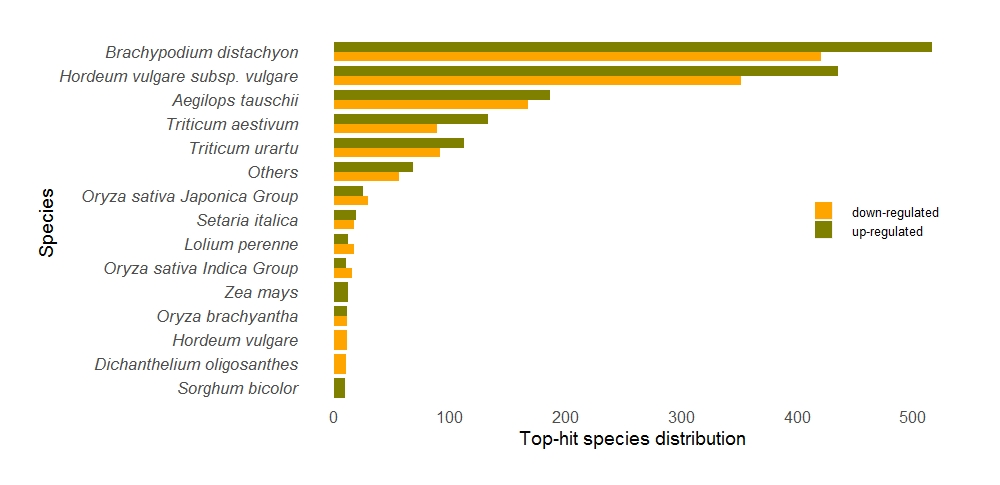

Supplement: Supplementary file 6 — Additional file 6: Fig S3. Top hit species distribution on the basis of sequence alignments and lowest E values. [file 13104_2019_4349_MOESM6_ESM.jpeg]
