# Supplementary material for: Transcriptome sequencing of Festulolium accessions under salt stress
Source: BMC Res Notes. 2019 May 31;12:311. doi: 10.1186/s13104-019-4349-2 (PMC6545024; doi:10.1186/s13104-019-4349-2)
Supplement: Supplementary file 3 — Additional file 3: Table S2. Summarized benching marking for each transcriptome assembly (C: complete [S: single, D: duplicated], F: fragmented, M: missing. [file 13104_2019_4349_MOESM3_ESM.pdf]

Species	Busco groups searched	Busco notation assessment results
Festulolium braunii (LM x FP)	1440	C:70.4%[S:19.3%,D:51.1%],F:14.4%,M:15.2%
Festulolium pabulare (LM x FA) *festucoid*	1440	C:67.9%[S:18.3%,D:49.6%],F:19.2%,M:12.9%
Festulolium pabulare (LM x FA) *festucoid*	1440	C:67.9%[S:16.9%,D:51.0%],F:14.9%,M:17.2%
Festulolium pabulare (LM x FA) *loloid*	1440	C:68.6%[S:19.0%,D:49.6%],F:19.1%,M:12.3%
